# Multiple neural control and stabilization

**DOI:** 10.1007/s00521-016-2659-z

**Published:** 2016-11-10

**Authors:** Fathi Fourati

**Affiliations:** Control and Energy Management Laboratory (CEM-Lab), University of Sfax, Sfax, Tunisia

**Keywords:** Behaviors, State variables, Multiple neural controllers, Stabilization

## Abstract

In this paper, we present a multiple neural control and stabilization strategy for nonlinear and unstable systems. This control strategy method is efficient especially when the system presents different behaviors or different equilibrium points and when we hope to drive the whole process to a desired state ensuring stabilization. The considered control strategy has been applied on a nonlinear unstable system possessing two equilibrium points. It has been shown that the use of the multiple neural control and stabilization strategy increases further the stability domain of the system further than when we use a single neural control strategy.

## Introduction

Multiple models characterizing different plant operation modes are used to predict the system behaviors. They are the one that best describes the plant and used to initialize new adaptation and/or generate new control input. From a practical point of view, the need to use multiple models in control is often necessary specially when sudden changes in the plant occur, in order to give better performance such as more accurate tracking and larger operation domain. In addition to the abilities of neural networks to imitate nonlinear plant characteristics, both multiple models and neural networks tools have attracted researchers to investigate in the domain of control of complex and nonlinear system especially the field of multiple neural control strategies.

In the 1960s and 1970s, most of works on the multiple model control were based on optimal control. Specifically, problem solving was based on the use of Kalman filters and linear control minimizing a quadratic loss function [1].

In the context of identification, there is no new means used in the multiple model approach, but in the control context, the switching problem was raised and the first proposals were published by Martensson [2]. Following, two types of switching will begin to appear in the literature. The first is known as direct switching, where the choice of the next controller is predetermined and depends on the outputs of the system. The second is known as indirect switching, where local models are used at each moment in which controller will be used [3]. The latter kind of switch is also called supervised control.

There has been a major research activity to extend the multiple model approach in the control field. Narendra et al. [4] presented a general methodology to design a multiple model adaptive control of uncertain systems. This methodology makes systems to operate effectively in an environment with a high degree of uncertainty. As applications, they considered a system described by various behaviors; each behavior is represented by a model including the dynamic relating to the considered environment.

In their book, Murray-Smith and Johansen [5] have made a collection of a number of articles on multiple model approaches. This book considers the various aspects and applications of multiple model approach for modeling, identification and control of nonlinear systems, and it summarizes the theory and application of multiple model adaptive control. In this book, the authors also open up horizons of research on the topic of adaptive multiple model control, such as the determination of the local models number of the complex system to be controlled, the choice of the types of controllers and the validity of the models (weighting functions).

Since 2000, research on multiple model approach has geared toward stability analysis and robust control of systems described by multiple models [6].

This paper is organized such that Sect. 2 presents a general description of a multiple neural control. Section 3 presents the structure of the multiple neural control and stabilization strategy. In Sect. 4, we show a single neural control stabilization method, and in Sect. 5, simulation results are carried out using the multiple neural control and stabilization on an unstable nonlinear system. Finally, a conclusion and prospects are given in Sect. 6.

## Multiple neural control

The application of multiple neural control strategy is based on neural models, which incorporates a set of pair model/controller. Combination and switching between models are the only characteristics of the multiple neural control [4, 5, 7, 8].

**Fig. 1:**
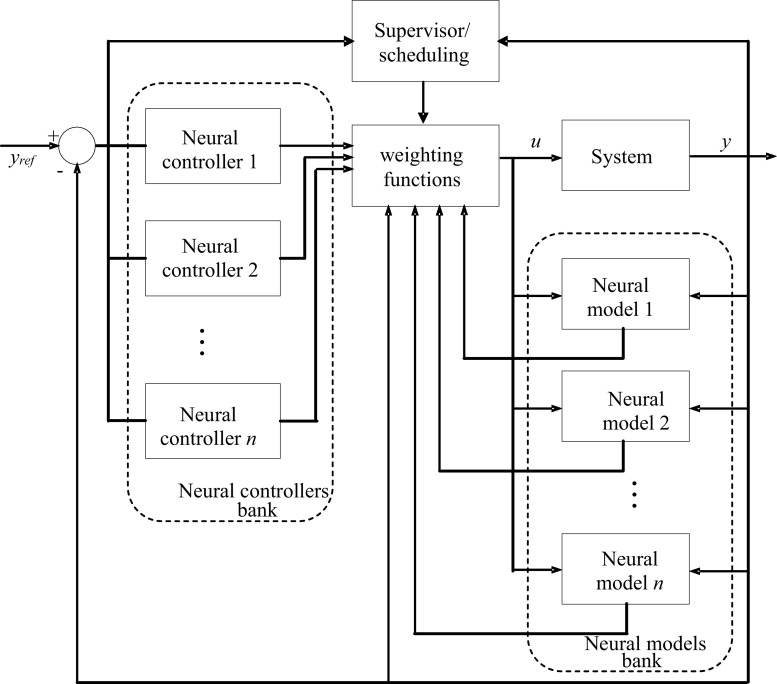
Multiple neural control strategy

The general outline of this control strategy is shown in Fig. 1.

The weighting function *f*_*i*_(*x*) (*i* ∈ {1,…, *n*}) represents the validity of the model number *i* (and/or the corresponding controller). In the case when we select a single controller at a given instant (e.g., the *i* ^ème^ controller), the value of the function *f*_*i*_(*x*) is equal to 1 and 0 for all others *f*_*j*_(*x*) (*j* ∈ {1,…, *n*} and *j*≠*i*). The value of the function *f*_*i*_(*x*) is belonging to the range [0, 1] in the case when we combine all models and controllers.

The banks of neural models and neural controllers are made after learning steps from sub-databases representing different behaviors of the controlled system.

## Multiple neural control and stabilization strategy

The multiple neural controller and stabilization strategy is used in order to increase the system’s stabilization domain. It consists on the build of neural controllers of which the learning step is carried out through sub-databases representing different regions (behaviors) of the system.

### Principle

**Fig. 2:**
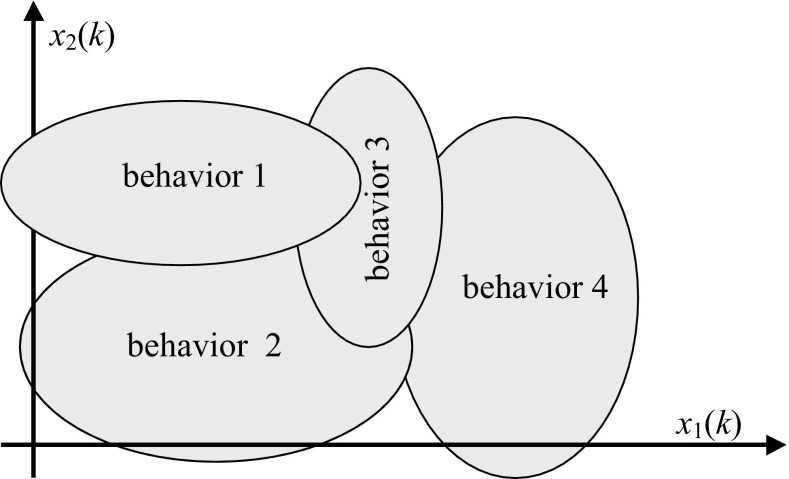
Behaviours partition in operating domain

The principle idea in this control strategy is to decompose the operating domain into locals regions where the system presents different behaviors (comportments in the case of equilibrium points) in order to solve modeling problems, control and stabilization [6, 9]. An example of behaviors partition in an overall operating domain is shown in Fig. 2.

The multiple neural control and stabilization strategy is based on the accomplishment of neural controllers direct neural models related to each behavior. The achievement of the learning for controllers and direct models is made from sub-databases identified around the center of each behavior. Each region may be represented in the space of a sphere or an ellipsoid whose center is the desired stabilization point.

### Structure of the multiple control and stabilization

**Fig. 3:**
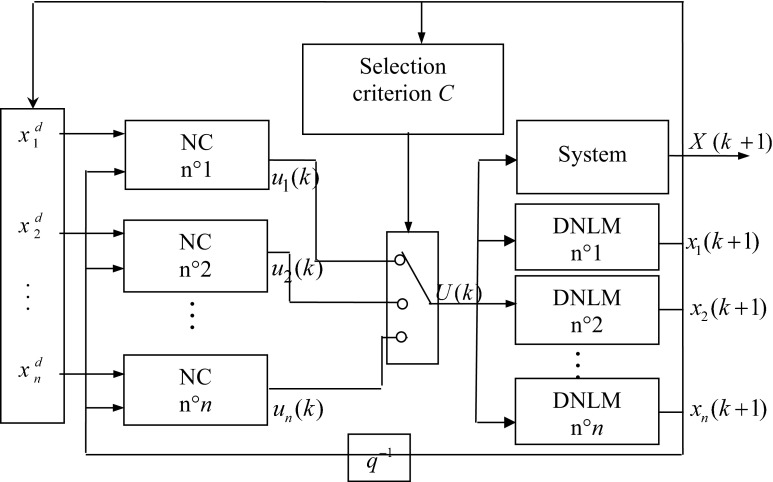
Multiple neural control and stabilization strategy

In our case, the centers of the operating modes (behaviors) are the equilibrium system points. The used controllers’ selection method is a binary one, so at each simple time only one neural controller (NC) from the bank of controllers is active. The selection criterion is based on the computation of algebraic distance between the current states of the direct neural local model (DNLM) and the desired state. The chosen controller is the one in which the corresponding direct neural local model gives the minimal distance. The diagram of the control strategy is illustrated in Fig. 3.

The selection criterion *C* (1) is based on the computation of the minimum distance between the current and the desired states, so the selected neural controller *i* is such that:

1 $$C = \hbox{min} (d_{i} )\quad i\; \in \left\{ {1,{ \ldots },\;n} \right\}$$

where $d_{i}$ is the distance between the current state $x_{i} (k)$ of the direct neural local model *i* and the desired state $x_{j}^{d}$.

### Controller principle

Consider a nonlinear system described by the following state-space model [9]:

2 $$x(k + 1) = f[x(k), \, u(k)]$$

where $x(k) \in R^{n}$ is the vector of state variables at time *k* $u(k) \in R^{m}$ is the control vector and *f* [.] is the vector of nonlinear functions.

It is assumed that the state variables are accessible and measurable. We can write (2) such that:

3 $$x(k + 2) = f[x(k + 1), \, u(k + 1)]$$

which can be written:

4 $$x(k + 2) = f[f[x(k), \, u(k)] \, , \, u(k + 1)]$$

This implies that the states at time *k* + 2 are determined from the states at time *k* and the control values between times *k* and *k* + 2.

By repeating this reasoning, we can write:

5 $$x(k + N) = f[{ \ldots }f[f[x(k), \, u(k)] \, , \, u(k + 1)] \, ,{ \ldots },u(k + N - 1)]$$

which can be rewritten in the following compact form:

6 $$x(k + N) = F[x(k),U(k)]$$

where

7 $$U(k)^{T} = [u(k), \, u(k + 1),{ \ldots },u(k + N - 1)]$$

In conclusion, the states at time *k* + *N* are determined by the state vector at time *k* and controls values between times *k* and *k* + *N* − 1.

If Eq. (6) is invertible [10], then *U*(*k*) can be solved according to *x*(*k* + *N*) and *x*(*k*). In fact, the authors proved that in this case, the condition of invertibility is interpreted as a local condition freely achievable. In addition, in Ref. [11], it is shown that if the linearized model of the system is controllable and observable, then the local inverse model of the system exists.

8 $$U(k) = G[x(k), \, x(k + N)]$$

where *G* is a nonlinear function and Eq. (8) is a fundamental relationship representing the inverse dynamic of the system [10]. The nonlinear function *G* can be approximated by a neural network. This last will be exploited as a neural controller providing the control actions to stabilize the system around an equilibrium point or a desired state.

### Structure of the neural controller

A large class of nonlinear dynamic systems is presented with the state-space models (2), so to realize neural control strategy it is necessary to build neural controllers based on input and states data.

**Fig. 4:**
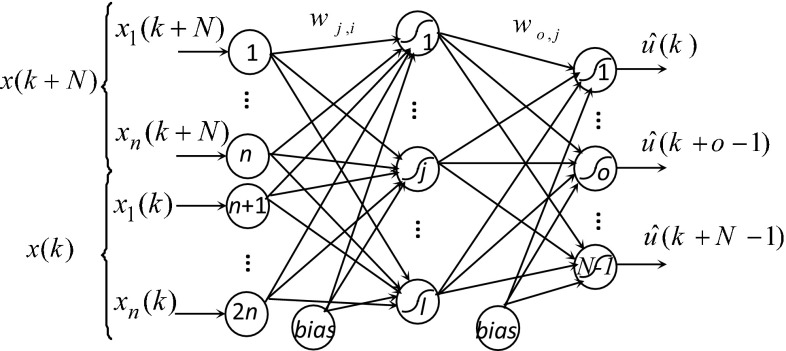
Structure of the neural controller

Generally, a multilayer feed-forward neural network with one hidden layer is used to model the direct and inverse dynamic nonlinear systems [10, 12, 13]. The same structure of neural network will be used to generate the system control law, given the current state and the future state (desired state) [14]. Figure 4 shows the structure of the used neural controller.

The connection weight *w*_*j,i*_ network and *w*_*o,j*_ are adjusted in order to minimize a quadratic error criterion (12) between network outputs *Û*(*k*) and the desired outputs *U*(*k*) [10–13].

The activation function used is sigmoidal one given by (13).

The neural controller output layer contains *M* = *N*·*m* nodes if the dimension of the control vector *u*(*k*) is equal to *m*.

*N* is the necessary number of iterations to evolve the system from the actual state *x*(*k*) to the future state *x*(*k* + *N*).

This neural network structure provides a function $\hat{G}[x(k),\;x(k + N),\;W]$ that models the inverse dynamics of the system. It is trained to provide the control action law *Û*(*k*) to the system.

9 $$\hat{U}(k) = \hat{G}[x(k), \, x(k + N), \, W]$$

*W* is the vector connection weights of the neural network.

The number of nodes in the input layer is defined according to the number of current and future states.

The number of nodes of the output layer is equal to the components number of the sequence of control actions applied to the system to reach the future state from the current state.

The number of nodes in the hidden layer is chosen after learning experiences. It is fixed by the structure which gives the lowest value of the error criterion (12).

### Learning procedure

**Fig. 5:**
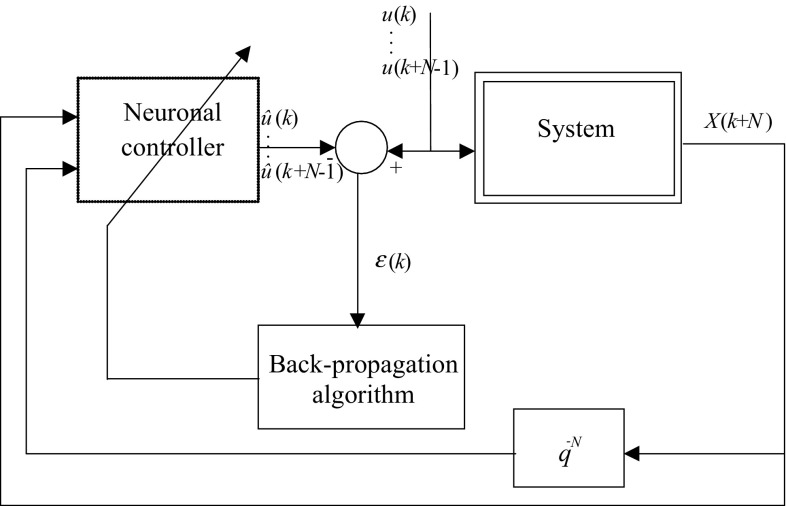
Learning procedure of the neural controller

The learning procedure of the neural controller is referred to the inverse neural modeling of the system [10–15] and [16]. The process that accomplished this step is shown in Fig. 5.

Here, sequences of synthesized signal *U*(*k*) are applied as inputs to the system. The corresponding states of these last are used as inputs to the neural network of which the outputs are compared with the training signal (the input of the system). The resulting error is used to adjust the neural network connection weights. This procedure tends to force the neural network to emulate the inverse dynamics of the system.

This learning structure is a classic method of supervised learning, where the teacher (i.e., the synthesis signal) provides directly target values to the output of the learner (i.e., the network model).

The used algorithm for learning is the back-propagation one ([17, 18]). For each input vector, the network calculates the output vector and adjusts the connections weights as described by Eqs. (16) and (20). The purpose of learning is to minimize the error *ε*(*k*) obtained for each learning pattern.

10 $$\varepsilon (k) = \sum\limits_{q = 1}^{N} {\sum\limits_{o = 1}^{m} {\left[ {u_{od} (k - q + 1) - u_{o} (k - q + 1)} \right]} }^{2}$$

### Weights connection adaptation

The values of the input nodes are distributed to the hidden nodes through the weights connection *w*_*ji*_. The input value of the *j*th node in the hidden layer is computed such that:

11 $${\text{net}}_{j}^{h} = \sum\limits_{i = 1}^{2n} {w_{ji} \, x_{i} } + \theta_{j}^{h}$$

where *w*_*ji*_ is the weight connection between *j*th node and the *i*th node of the input layer and *θ*_*j*_ is the bias value of the node *j*.

The output of the node *j* is given by:

12 $$e_{j} = g\left( {{\text{net}}_{j}^{h} } \right)$$

where *g* is a sigmoidal function (13)

13 $$g(x) = \frac{1}{{1 + e^{{ - x_{j} }} }}$$

The input value of the *o*th node in the output layer is computed such that

14 $${\text{net}}_{o}^{s} = \sum\limits_{j = 1}^{L} {w_{o,j} \, e_{j} } + \theta_{{_{o} }}^{s}$$

the output is:

15 $$s_{o} = g\left( {{\text{net}}_{o}^{s} } \right)$$

The adaptation of the connection weights *w*_*oj*_ between the hidden layer and the output layer is performed as follows:

16 $$w_{o,j} [t + 1] = w_{o,j} [t] - \eta \frac{\partial \, \varepsilon }{{\partial \, w_{o,j} }}$$

17 $$\frac{\partial \, \varepsilon }{{\partial \, w_{o,j} }} = \frac{\partial \, \varepsilon }{{\partial \, u_{o} (k)}}\frac{{\partial \, u_{o} (k)}}{{\partial \, w_{o,j} }}$$

18 $$\frac{\partial \, \varepsilon }{{\partial \, u_{o} (k)}} = - (u_{od} (k) - u_{o} (k))$$

and

19 $$\frac{{\partial \, u_{o} (k)}}{{\partial \, w_{o,j} }} = s_{o}^{'} \cdot e_{j} = \frac{{e^{{{\text{net}}_{o}^{s} }} }}{{\left( {1 + e^{{{\text{net}}_{o}^{s} }} } \right)^{2} }}\frac{1}{{\left( {1 + e^{{ - {\text{net}}_{j}^{h} }} } \right)}}$$

The adaptation of the connection weights *w*_*ji*_ between the input layer and the hidden layer is such that:

20 $$w_{j,i} [t + 1] = w_{j,i} [t] - \eta \frac{\partial \, \varepsilon }{{\partial \, w_{j,i} }}$$

21 $$\frac{\partial \, \varepsilon }{{\partial \, w_{j,i} }} = \frac{\partial \, \varepsilon }{{\partial \, u_{o} (k)}}\frac{{\partial \, u_{o} (k)}}{{\partial \, w_{j,i} }}$$

and

22 $$\frac{{\partial \, u_{o} (k)}}{{\partial \, w_{j,i} }} = \sum\nolimits_{o = 1}^{N \cdot m} {s_{o}^{'} .w_{o,j} \cdot e_{j}^{'} \cdot x_{i} }$$

where

23 $$s_{o}^{'} (x) = \frac{{\partial \;s_{o} (x)}}{\partial \, x} = \frac{{e^{{{\text{net}}_{o}^{s} }} }}{{\left( {1 + e^{{{\text{net}}_{o}^{s} }} } \right)^{2} }}$$

24 $$e^{'} (x) = \frac{\partial \, e(x)}{\partial \, x} = \frac{{e^{{{\text{net}}_{j}^{h} }} }}{{\left( {1 + e^{{{\text{net}}_{j}^{h} }} } \right)^{2} }}$$

where *η* is the learning rate.

## Single neural stabilization strategy

**Fig. 6:**
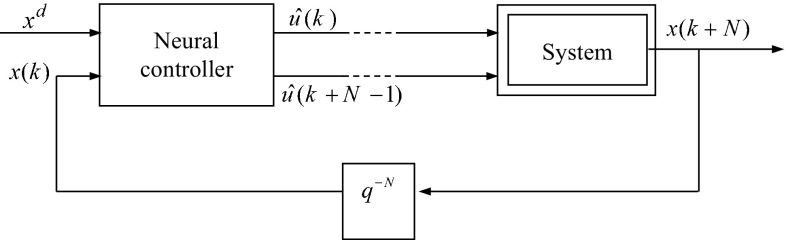
Single neural control and stabilization strategy

After a learning step, the neural network emulating the inverse dynamic of the system can be operated in a closed-loop control providing the control law. In this case, the neural network is placed in a cascade with the system. Both of them (neural network and system) established a neuronal stabilizing feedback control states [14]. Figure 6 shows this control structure.

The controller provides the system an *N* control values in each interval of *N* sampling periods.

The parameter *N* must be at least equal to *n* (order system) for local controllability [11].

This structure of stabilization and control will be used in the case of multiple neural control and stabilization.

**Fig. 7:**
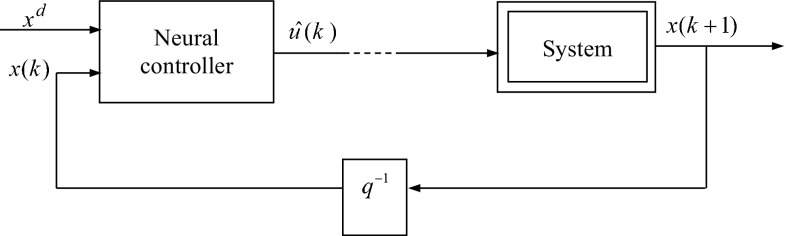
Modified single neural control and stabilization strategy

In order to compute the value of the selection criterion (1) at each sample time in the case of multiple neural control and stabilization, only the first component of the input vector $\hat{u}(k)$ will be applied to the system. This is illustrated in Fig. 7.

## Simulation results

In this section, we present the simulation results after the application of multiple neuronal and stabilization strategy for a nonlinear unstable system extract from Ref. [19].

### Presentation of the system

The considered nonlinear system of the third order (three states) defined by the following equations:

25 $$\left\{ \begin{array}{l} x_{1} (k + 1) = x_{2} (k) - x_{3} (k) + u^{2} (k) \hfill \\ x_{2} (k + 1) = 2x_{1} (k) - [1 + 0.5x_{2} (k)] \cdot u(k) \hfill \\ x_{3} (k + 1) = x_{1} (k) \, [x_{2} (k) - x_{3} (k)] + u(k) \hfill \\ \end{array} \right.$$

This system presents two equilibrium points $$x_{e1}^{T} = \left( {\begin{array}{*{20}c} 0 & 0 & 0 \\ \end{array} } \right)$$ and $$x_{e2}^{T} = \left( {\begin{array}{*{20}c} 1 & 2 & 1 \\ \end{array} } \right)$$.

In order to apply the multiple neural control and stabilization strategy, we have made two sub-databases around each equilibrium point (behavior). These sub-databases will be used to learn neural controllers and direct neural local models useful to compute the selection criterion *C* and then to select the appropriate controller.

### Sub-databases

The considered system (25) is highly unstable and divergent. The sub-databases are built by varying the system input at a random manner. The inputs should be bounded with low values, so that the system doesn’t diverge from the first iterations. For both sub-databases, we adopt an input signal $u(k) \in [ - 0,5;\;0,5]$ and different initial states *x*(*k*) chosen near a considered equilibrium point. The future states values *x*(*k* + *N*) are the computed, and all values from time *k* to time *k* + *N* (in our case *N* = 3) are then recorded in text file establishing a sub-database which will be used to learn neural controller and direct neural local model.

For the first sub-database related to the first equilibrium point $$x_{e1}^{T} = \left( {\begin{array}{*{20}c} 0 & 0 & 0 \\ \end{array} } \right)$$, the initial states $$x(k) = \left( {\begin{array}{*{20}c} {x_{1} (k)} & {x_{2} (k)} & {x_{3} (k)} \\ \end{array} } \right)^{T}$$ are chosen at random values belonging to the interval $[ - 1,1]$ and the second sub-database on the second equilibrium point $$x_{e2}^{T} = \left( {\begin{array}{*{20}c} 1 & 2 & 1 \\ \end{array} } \right)$$ where the initial states are chosen at random values belonging, respectively, to the intervals $[0,2]$, $[1,3]$, $[0,2]$.

### System response with multiple neural control and stabilization

In this part, we will apply the multiple neural controller and stabilization strategy in order to stabilize the system around its two equilibrium states which are defined as desired states. The controller’s selection is based on the computation result of the criterion (1) (here *n* = 2), minimal distance between the current and the desired states.

26 $$x_{1}^{d} = x_{e1}^{T} = \left( {\begin{array}{*{20}c} 0 & 0 & 0 \\ \end{array} } \right)\;{\text{and }}x_{2}^{d} = x_{e2}^{T} = \left( {\begin{array}{*{20}c} 1 & 2 & 1 \\ \end{array} } \right)$$

**Fig. 8:**
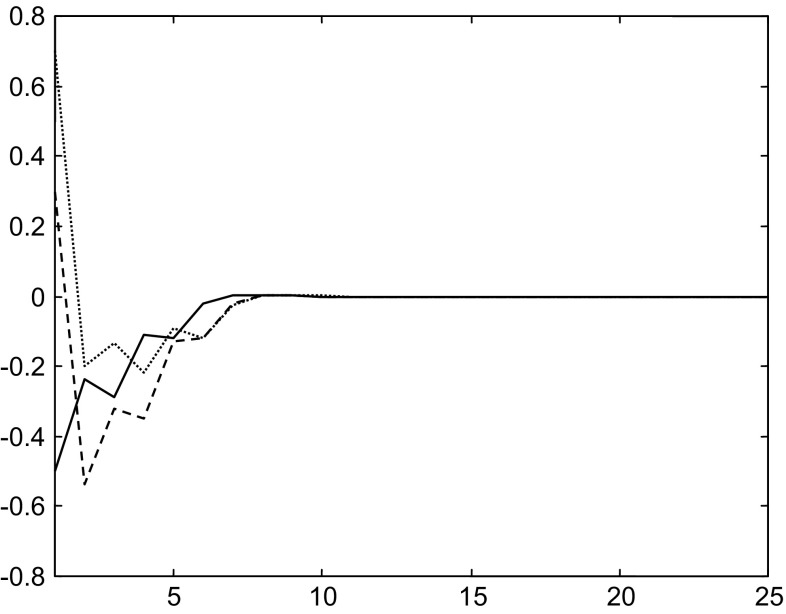
Evolutions of the states in the case of the multiple neural control and stabilization strategy with the initial state $x_{i} (1)$

To show the benefits of the considered control and stabilization strategy, we have considered initial states in the neighborhood of the first equilibrium point and others near the second equilibrium point, and then, we have applied this strategy of control and stabilization.With a first initial state $$x_{i} (1) = \left( {\begin{array}{*{20}c} { - 0.5} & {0.3} & {0.7} \\ \end{array} } \right)^{T}$$, the states evolutions through time with the multiple neural control and stabilization strategy are shown in Fig. 8.

Here, the first controller is selected and the system stabilizes around the desired state (first equilibrium point) $x_{1}^{d}$. In this case, the distance between initial and desired states is $d_{1} = 0.911$. With the second controller where the distance between initial and second desired states is $d_{2} = 2.287$, the system diverges.

**Fig. 9:**
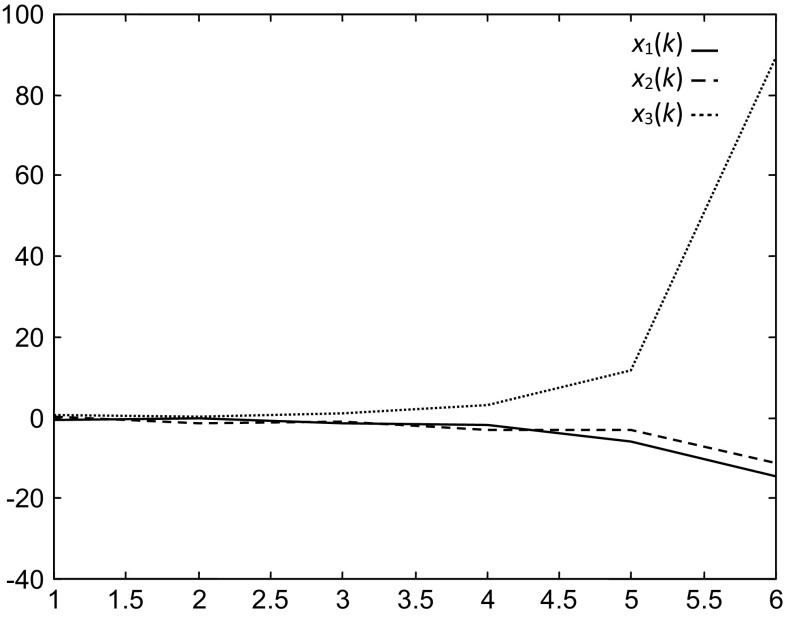
Evolutions of the states using single neural control stabilization strategy with the initial state $x_{i} (1)$

With the same initial state and using single neural control stabilization, the states diverge. Figure 9 shows the evolution of the states.

Note that with the multiple neural control strategy, the system is stabilized around the first equilibrium point (desired state), whereas with a single neural control strategy the system diverges.

**Fig. 10:**
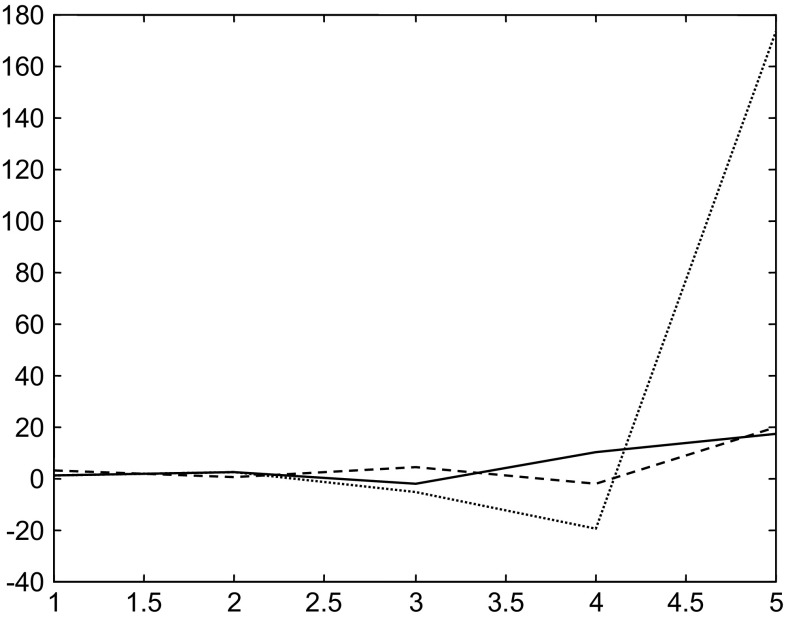
Evolutions of the states using single neural control stabilization strategy with the initial state $x_{i} (2)$

Then with a second initial state is $$x_{i} (2) = \left( {\begin{array}{*{20}c} 1 & {3.3} & 1 \\ \end{array} } \right)^{T}$$, the evolutions of the states through time with a single neural control strategy are given in Fig. 10.

We remark that with the single control strategy the states diverge.

**Fig. 11:**
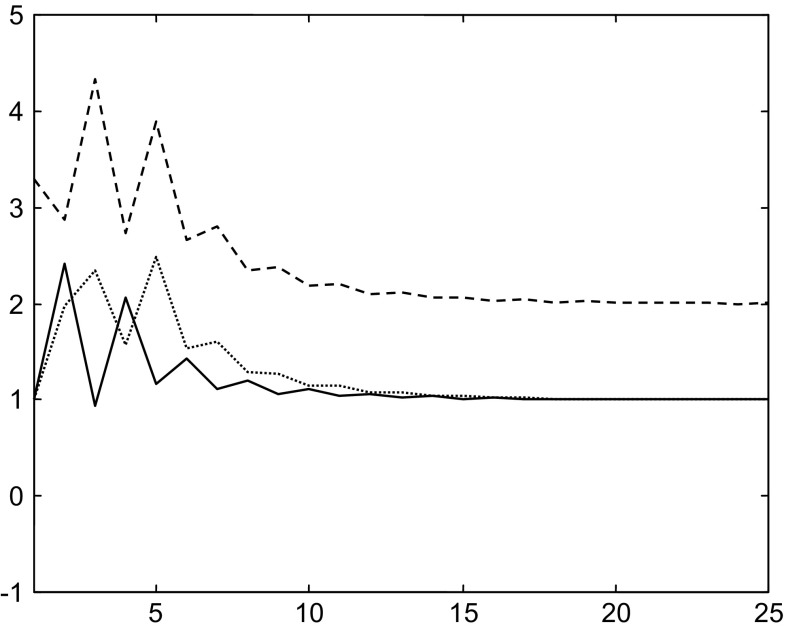
Evolutions of the states in the case of the multiple neural control and stabilization strategy with the initial state $x_{i} (2)$

With the same initial state and using multiple neural control strategy, the states stabilize around the second equilibrium point. Figure 11 shows the evolution of the states.

Here, the selected controller is the second one, the distance between initial and desired states is $d_{2} = 1.3$, and the system stabilizes around the desired state (second equilibrium point) $x_{2}^{d}$. While with the first controller where the distance between initial and desired states is $d_{1} = 3.59$, the system diverges.

These results confirm the efficient of the multiple neural control and stabilization strategy.

## Conclusion

We have presented a multiple neuronal control and stabilization strategy which can be applied on systems characterized by different behaviors in different regions of the functional domain. In the simulation results, we have considered an unstable system possessing two equilibrium points. So two neural controllers and two direct neural local models have been built around each point; then, the multiple neural control and stabilization strategy has been applied using a distance criterion between the current and the desired states to select the appropriate controller. The use of the latter strategy increases the system stabilization domain further. As future work, we hope to use an adaptive multiple neural control and stabilization strategy with weighting functions representing the validity of models.
